# Pulmonary and Cerebral Fat Embolism Syndrome After Total Knee Replacement

**DOI:** 10.4021/jocmr1251w

**Published:** 2013-04-23

**Authors:** Soo Hyun Yeo, Hyuk Won Chang, Sung Il Sohn, Chul Hyun Cho, Ki-Cheor Bae

**Affiliations:** a Department of Radiology, Keimyung University School of Medicine, Korea; b Department of Neurology, Keimyung University School of Medicine, Korea; c Department of Orthopaedic Surgery, Keimyung University School of Medicine, Korea

**Keywords:** Fat embolism syndrome, Tomography, X-Ray Computed, Magnetic Resonance Imaging, Diffusion magnetic resonance imaging

## Abstract

Fat embolism occurs after long bone fracture or orthopedic surgery and usually shows mild symptom. But it rarely results in fat embolism syndrome, presenting as multiorgan dysfunction such as lung, brain and skin. Although the diagnosis of fat embolism syndrome is mostly based on clinical features, we experienced fat embolism syndrome involving lung and brain, showing typical imaging findings in pulmonary computed tomography and brain magnetic resonance image. So we present interesting case about fat embolism syndrome after total knee replacement with reviewing associated literatures including imaging findings.

## Introduction

Fat embolism is a relatively common complication after pelvic and long bone fracture, and is commonly seen after procedures or conditions such as orthopaedic surgery, severe burns, liver injury, closed-chest cardiac massage and liposuction [1-3]. In general, fat embolism is asymptomatic, but in 1-5% of patients, fat embolism results in the fat embolism syndrome associated with multiorgan dysfunction involving sites such as the lung, brain and skin due to direct entry of fat globules into the systemic circulation [1, 2, 4].

Fat embolism syndrome typically develops 24 to 72 hours after the initial injury and usually appears with a classical triad consisting of hypoxemia, neurologic abnormality and petechial rash [2].

Fat embolism syndrome with either pulmonary or cerebral involvement confirmed by radiologic modalities have been described in many previous studies, but few radiologic reports have described combined pulmonary and cerebral fat embolism in a patient with fat embolism syndrome after trauma or orthopaedic surgery [1, 2, 5-7]. So, we present an interesting case of fat embolism syndrome involving both brain and lung after total knee replacement, with a review of the literature including typical imaging findings.

## Case Report

A 64-year-old woman visited our orthopaedic department for pain in both knees.

**Figure 1:**
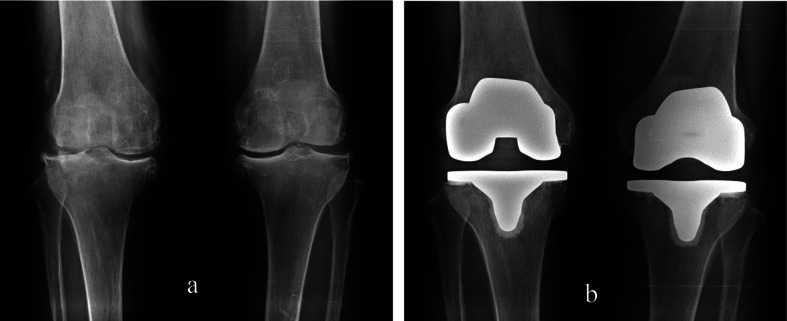
(a) Preoperative both knee radiographs reveal narrowing of articular space with subchondral sclerosis and spur formation. (b) Postoperative both knee radiographs reveal metallic prosthesis in both knee joints.

The patient had suffered from pain in both knees for six years and the patient was managed with medications for pain control at a local medical centre. However, the pain was more aggravated from six months prior, and the medication was not sufficient to control the pain. After the patient had visited our orthopaedic department, the patient was diagnosed as having osteoarthritis in both knees and total knee replacement was recommended (Fig. 1a).

The patient was relatively healthy without any previous medical or surgical history except for hypertension. The patient had a limitation of motion in both knee joints, but there were neither external wounds nor sensory changes in both lower extremities.

Preoperative examinations such as routine laboratory tests and imaging studies such as chest X-rays and a pulmonary function test were within normal limits. The patient underwent total knee replacement surgery in both knee joints (Fig. 1b).

**Figure 2:**
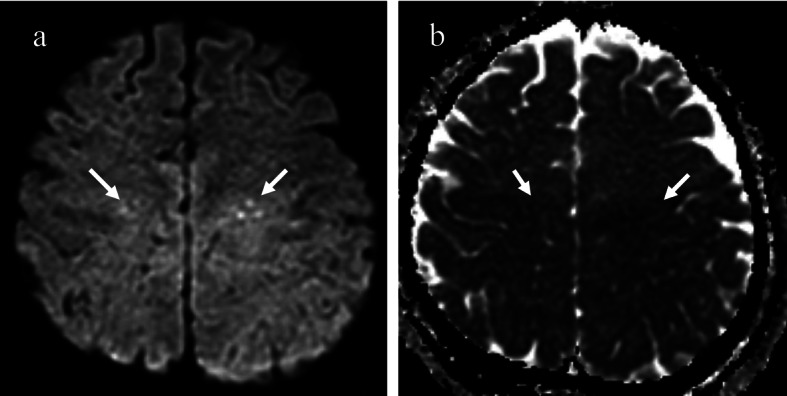
(a) A diffusion weighted image of the brain reveals multiple small dot-like high signal intensities in both centrum semiovale (white arrows). (b) An apparent diffusion coefficient (ADC) map shows a decreased ADC at the lesions (white arrows).

On the second postoperative day, the patient demonstrated stuporous mentality with eyeball deviation and became markedly dyspnoeic and hypoxemic. Arterial blood gas analysis showed both a decreased PO_2_ as 33.9 mmHg and an oxygen saturation level of 68.4%. Brain MR and pulmonary CT imaging were performed to exclude cerebral and pulmonary fat embolism. Brain MRI demonstrated multiple small dot-like high signal intensities in both the centrum semiovale and subcortical white matter on diffusion-weighted imaging (DWI), which was suggestive of cerebral fat embolism (Fig. 2).

**Figure 3:**
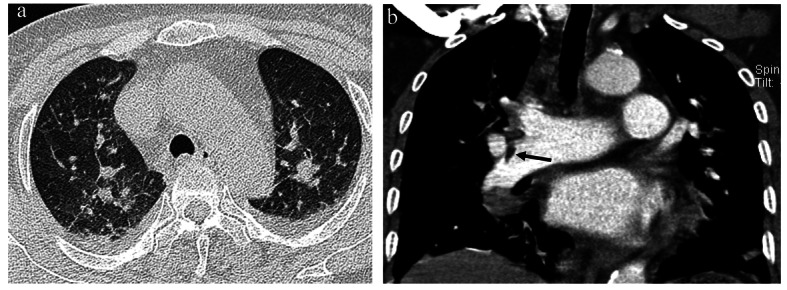
(a) A high resolution CT image of the lung reveals patchy ground glass opacities in both lung fields and bilateral pleural effusion. (b) A coronal reformatted image of the pulmonary artery shows a small filling defect in the right intermediate branch of the pulmonary artery and the mean attenuation value of the lesion is -42 Hounsfield units (HU), suggesting the presence of fat embolism (black arrow).

High resolution CT of the lung demonstrated the presence of patchy ground glass opacity in both lungs and bilateral pleural effusion. An enhanced chest CT image demonstrated the presence of a small embolus in the right main pulmonary artery. The mean attenuation value of the embolus was -42 Hounsfield units (HU), suggestive of fat embolism (Fig. 3).

The patient was treated supportively with mechanical ventilation, supplemental oxygen and heparinization. The patient showed gradual improvement of the respiratory and neurological status and no further complications were noted such as wound infection or rebleeding. The patient was discharged from hospital with nearly a fully recovered state two weeks after surgery.

## Discussion

Fat embolism syndrome is a clinical diagnosis and is caused by the release of fat droplets into the systemic circulation after conditions or procedures such as pelvic or long bone fracture, orthopaedic surgery or other interventions [1, 4].

The pathophysiology of fat embolism syndrome is unclear, but two mechanisms have been suggested [3]. One mechanism is a mechanical mechanism that suggests that physical obstruction of the systemic vasculature occurs that is caused by fat globules released from the bone marrow of long bones due to increased intramedullary pressure after trauma or orthopaedic surgery such as placement of intramedullary rods [3, 4, 8]. The other mechanism is a biochemical mechanism that suggests that free fatty acid can cause a local inflammatory response and direct toxicity to the lung or capillary endothelium. Fat emboli are metabolised to free fatty acids; in addition, different hormonal changes after trauma or sepsis can induce systemic circulation of free fatty acids [3, 4]. The conversion of fat emboli to free fatty acids requires 24 to 48 hours; thus, symptoms of fat embolism syndrome occur at 24 to 72 hours after the initial insult.

Fat embolism syndrome is usually manifested as a multiorgan disorder that typically involves the respiratory system, central nervous system, cardiovascular system, skin and eyes. Gurd and Wilson have suggested criteria for the diagnosis of fat embolism syndrome, with three major criteria including respiratory insufficiency, cerebral involvement, petechial rash and nine minor criteria such as pyrexia, tachycardia, retinal changes, jaundice, renal change, anemia, thrombocytopenia, increased erythrocyte sedimentation rate (ESR) and fat macroglobulinemia [9]. These investigators have suggested that at least two symptoms for the major criteria or one symptom for the major criteria and four symptoms for the minor criteria must be present to diagnose the syndrome.

The diagnosis of fat embolism syndrome is mainly based on clinical criteria but further confirmation can be achieved by finding a typical image pattern on CT or MR images. Pulmonary CT or brain MR imaging has been used to determine the etiology of a sudden change of mentality or the presence of respiratory symptoms.

Common CT findings of pulmonary fat embolism are non-specific focal or diffuse consolidation and ground glass opacity that may be due to interstitial or alveolar haemorrhage, oedema and chemical pneumonitis [2]. A finding of a filling defect in the pulmonary artery that shows negative HU is more favourable for a diagnosis of pulmonary fat embolism [2, 8].

MRI is the most sensitive technique to confirm the presence of a cerebral fat embolism, particularly the use of a diffusion-weighted imaging (DWI) sequence at an acute stage [5, 6]. Typical findings of cerebral fat embolism are multiple small, dot-like hyperintense lesions on DWI and T2-weighted images, situated within the periventricular and deep white matter [6]. The number and size of the lesions are usually correlated with the degree of neurologic disability as measured by the Glasgow Coma Scale [6].

To the best of our knowledge there is only one case report that has described fat embolism syndrome involving the lung and brain with radiological findings, except one case report [7]. The paucity of these cases is probably due to the infrequent use of CT or MR imaging in the past. However, with increased use of CT or MR imaging to confirm the presence of fat embolism syndrome and to exclude other causes, it is possible that orthopaedic surgeons will more commonly encounter fat embolism syndrome involving the brain, lung and other sites after trauma or orthopaedic surgery.
